# Beyond Diagnosis: Evolving Prostate Biopsy in the Era of Focal Therapy

**DOI:** 10.1155/2011/386207

**Published:** 2010-12-09

**Authors:** J. L. Dominguez-Escrig, S. R. C. McCracken, D. Greene

**Affiliations:** Urology Department, Sunderland Royal Hospital, Kayll Road, Sunderland SR4 7TP, UK

## Abstract

Despite decades of use as the “gold standard” in the detection of prostate cancer, the optimal biopsy regimen is still not universally agreed upon. While important aspects such as the need for laterally placed biopsies and the importance of apical cancer are known, repeated studies have shown significant patients with cancer on subsequent biopsy when the original biopsy was negative and an ongoing suspicion of cancer remained. Attempts to maximise the effectiveness of repeat biopsies have given rise to the alternate approaches of saturation biopsy and the transperineal approach. Recent interest in focal treatment of prostate cancer has further highlighted the need for accurate detection of prostate cancer, and in response, the introduction of transperineal template-guided biopsy. While the saturation biopsy approach and the transperineal template approach increase the detection rate of cancer in men with a previous negative biopsy and appear to have acceptable morbidity, there is a lack of clinical trials evaluating the different biopsy strategies. This paper reviews the evolution of prostatic biopsy and current controversies.

## 1. Introduction

Prostate cancer is now recognized as one of the major medical problems facing the male population. In Europe, with an annual incidence of 214 cases per 1000 men, prostate cancer represents the most common solid tumour affecting men, having surpassed lung and colorectal cancers [1], and it is the second most common cause of cancer death in men [2].

 In 2008, an estimated 186,320 men were diagnosed with prostate cancer and 28,600 were expected to die from the disease in the USA [2].

Despite the rapid development of imaging and extensive clinical evaluation of PSA and its derivates, as well as novel prostate cancer biomarkers, prostate biopsy remains up to this day the only diagnostic test for the detection of cancer. However, far from a standardized practice, prostate biopsy is still evolving. Indeed, the ideal technique, the so-called biopsy “gold standard”, is still to be fully defined.

## 2. Limitations of Sextant Biopsy

Following the landmark study of Hodge and colleagues in 1989 [3], demonstrating the superiority of systematic TRUS biopsies compared to digitally directed sampling, TRUS-guided biopsies became the accepted standard for diagnosis of prostate cancer. In this scheme, 6 parasagital cores (biopsies from apex, middle, and base of each lobe) were obtained in a systematic randomized fashion. However, sampling was clearly limited, and subsequent studies have demonstrated a high false-negative rate of between 15 and 31% [4–7].

In an *ex vivo* model, where 90 patients undergoing radical prostatectomy for clinically localized disease underwent sextant biopsy immediately after removal of the gland, Svetec et al. demonstrated a 45.6% false-negative rate [8].

In the same year, Rabbani et al. studied the incidence and clinical significance of false-negative sextant biopsies in 118 patients with proven cancer, who underwent repeat sextant prostate biopsy before prostatectomy. Sextant biopsies were negative in 27/118 (23%) cases, all of these representing significant cancers [4].

Stochastic computer simulation models of ultrasound-guided biopsies, using mathematically reconstructed radical prostatectomy specimens, developed at the M.D. Anderson Cancer Center [9] and the University of Colorado [10], consistently demonstrated false-negative biopsy rates of 27% and 26.8%, respectively. Two main conclusions were drawn from these studies: first, sampling limitations by standard sextant biopsies missed “significant” prostate tumours; second, improved biopsy schemes for detection of low-volume cancers should include prostate gland volume and tumour distribution. In particular, biopsies of the transition zone (TZ), midline peripheral zone (PZ), and inferior portion of the anterior horn of the peripheral zone were highlighted, as evidenced by the 96% cancer detection rate for tumour volumes >0.5 cc when a 10-core biopsy scheme incorporating these areas was implemented [9]. 

In the light of the evidence from these and other similar studies [5, 11], the nineties saw a move away from the sextant biopsy, no longer considered as the “gold standard” for prostate cancer diagnosis. In an attempt to decrease false-negative rates, new approaches rapidly emerged: (a) in parallel to the technological improvements with modern US, more accurately targeted biopsies were assessed; (b) to avoid sampling limitations, number of cores rapidly increased; finally, (c) anatomical considerations guided the development of new biopsy schemes incorporating more lateral biopsies as well as the transitional and apical zones.

## 3. Limitations of Targeted Biopsies

Rapid technological advances in ultrasound (US) imaging, in particular the introduction of the 7.5 MHz probe in 1980, allowed a more accurate description of the prostate anatomy, with a much clear definition of the transitional-peripheral zone interface and made possible a more selective targeting of intraprostatic lesions. 

In 1997, Norberg et al. evaluated the sensitivity of targeted biopsies and compared it with the standard sextant protocol in 512 consecutive patients with suspected cancer. Patients had 8 or 10 standardized biopsy samples depending on the size of the gland. Additional targeted biopsies were taken from hypoechoic or hyperechoic lesions. Sensitivity was 59% for focal lesions detected by TRUS, 85% to 97% for different combinations of systematic biopsy samples, and 93% to 98% for a combination of systematic and targeted biopsies. Whereas the sensitivity for the standard sextant protocol was 85%, the addition of targeted biopsies increased the sensitivity to 93% [6]. Further evidence for a targeted approach has been reported by other contemporary studies [12, 13]. 

Toi et al. reported results on 7,426 transrectal ultrasound-directed biopsies performed at the Princess Margaret Hospital (Toronto, Canada). Patients underwent systematic biopsy with additional sampling of visible suspicious lesions. Overall, cancer detection rate was 43.9%. The presence of a sonographic lesion increased the likelihood of cancer detection (57.8% versus 30.8%). Targeted biopsies from these lesions had a significantly greater median percent of the core involved with cancer (50% versus 10%, *P* < .001) and grade (presence of Gleason ≥7 in 69.3% versus 28.3%, *P* < .001) [14]. 

Lee et al. enrolling 350 patients with ultrasound detected lesions, evaluated the diagnostic efficacy of TRUS-guided targeted prostatic biopsies and proposed a new scoring system for the prediction of malignancies, based on lesion characteristics depicted on TRUS. Utilizing this scoring system for cancer detection, the authors reported a positive predictive value of 80% when applied to the test set [13]. 

Evaluating the significance of suspicious lesions at TRUS, Shim et al. studied a cohort of 1,009 men undergoing biopsy for suspected cancer. Overall, cancer detection rate was 26.3%, being higher in patient with suspicious lesions (33.2% versus 21.5%, *P* < .001). The positive predictive value of additional lesion-directed biopsy was 18%. However, patients who had positive cores on lesion-directed biopsies, all were also found to have positive cores on random biopsies, and no patient had positive cores only on lesion-directed biopsies, questioning the real value of these schemes [15]. Supporting those findings, Onur et al. prospectively studied 3,912 patients undergoing sextant plus targeted biopsies of hypoechoic lesions (68%). The authors concluded that despite the higher prevalence of cancers discovered in prostates with hypoechoic areas, the hypoechoic lesion itself was not associated with increased cancer prevalence compared with biopsy cores from isoechoic areas [16]. Further contemporary studies, as well as recent evidence, question the value of targeted biopsies, which can at least be partly explained by the low specificity of ultrasound in characterising prostatic lesions [17]. The classic hypoechoic area in the peripheral zone is not always necessarily present [18]. Moreover, Ellis and coworkers noted that 37.6% of their detected cancers were diagnosed in isoechoic areas of the prostate [19]. A strategy of performing biopsy of only hypoechoic sectors will miss 24.6% of patients with prostate cancer [19]. A recent study by Spajic et al. in a cohort of 200 patients undergoing TRUS for clinically suspected cancer reported an incidence of isoechoic, hypoechoic, and hyperechoic lesions of 49%, 41.5%, and 9.5%, respectively. There was an overall cancer detection rate of 33%. Among patients with cancer, isoechoic, hypoechoic, and hyperechoic lesions were present in 31.8%, 60.6%, and 7.6%, respectively. Interestingly, the Gleason score of hyperechoic cancers was higher when compared with isoechoic and hypoechoic cancers [20].

## 4. Increasing the Number of Cores: Extended Protocols

With the obvious sampling limitations of the original sextant biopsy protocol, clinicians optimized biopsy schemes by increasing the number of cores. Intuitively, one would expect that increasing the sample size and including areas not sampled by the standard 6-core scheme would increase the cancer detection rate. 

An *ex vivo* study by Fink and coworkers, comparing sextant versus 10-core biopsy template in 91 prostatectomy glands demonstrated a higher detection rate for both initial (78% versus 60%) and repeated (90% versus 75%) biopsies, with the 10-core scheme [21]. 

Many published studies and literature reviews have demonstrated this presumption, giving further evidence of the importance of sampling the lateral and apical peripheral zones [22, 23]. Such extended schemes ranging from 10 to 18 cores have consistently reported cancer detection rates in the order of 40% by complementing the standard sextant template with added cores directed to the far lateral and mid-regions of the gland [24, 25], the base and middle of each lobe [26, 27], anterior TZ, middle PZ, and anterior horn [28].

In a study of 396 consecutive patients undergoing standard sextant plus laterally directed peripheral zone biopsies, Gore et al. reported a cancer detection rate of 40.4%. Furthermore, subset analysis of different core combinations demonstrated that a 10-core scheme including laterally directed cores at the base, mid-gland, and apex with midlobar base and apical sampling detected 98.5% of cancers [24]. 

In 2006, Eichler and colleagues published a systematic review and meta-analysis of published studies with a total of 20,698 patients. Data was pooled from 68 studies comparing a total of 94 extended schemes with the sextant standard, demonstrating once more that laterally directed cores significantly increase the diagnostic yield. In particular, 12-core with additional laterally directed cores detected 31% more cancers than the sextant scheme, with a comparable complication rate. Interestingly, no further benefit was seen in extended schemes with 18 to 24 cores, whereas the adverse event profile was poor with these templates. The authors concluded that more than 12 cores added no significant benefit [29].

In agreement with Eichler's meta-analysis, two recently published studies critically reviewing the optimal biopsy strategies have further supported the use of extended 10- to 14-core biopsy schemes as the new “gold standard” for first-time prostate biopsy [30, 31].

Nevertheless, these and other contemporary published studies have also highlighted the need for more intensive strategies in patients undergoing repeated biopsies. In this setting, despite of extended schemes, prostate biopsy is still associated with significant “false-negative” results as evidenced by the significant percentage of men with persistent clinical suspicion, who will be diagnosed at subsequent biopsy (range: 18.8 to 55.5%) [32–36].

## 5. Indications and Optimal Scheme for Rebiopsy

In spite of the evidence for rebiopsy in patients with previous benign histology and persistent clinical suspicion, the indications, the required number of cores, and even more so, the optimal biopsy scheme remain controversial. 

Supported by clinical evidence and endorsed by the AUA and the EAU, it is common practice to offer repeated biopsy to patients with initial negative biopsy and persistent clinical suspicion of cancer, dictated by abnormal DRE, persistent elevation of PSA, and initial histology. However, better understanding of the cancer biology and clinical significance of high-risk lesions (HGPIN and ASAP), as well as rapid changes in the management of patients with prostatic malignancy, particularly organ-confined disease, has dramatically widened the spectrum.

### 5.1. ASAP

Atypical small acinar proliferation (ASAP), a suspicious small focus of atypical glands, but with insufficient architectural and/or cytological criteria for definitive diagnosis [37], is found in approximately 5% of first-time prostate biopsies, and it has been associated with positive second biopsies in approximately 40% of cases [38]. In a recent study of patients with ASAP at initial 10- or 12-core biopsy, Scattoni and coworkers have reported a cancer detection rate of 39%, 35%, and 21% at first, second, and third rebiopsy, respectively. When HGPIN was associated with ASAP (17%), the cancer detection rate was higher (50%) [39].

### 5.2. HGPIN

While low-grade prostatic intraepithelial neoplasia (PIN) is not reported, the presence of isolated high-grade PIN (HPIN) should be reported in all biopsy specimens [40]. High-grade prostatic intraepithelial neoplasia (HGPIN) is characterized by architecturally normal ducts and acini lined with abnormal cells with prominent nucleoli and nucleomegaly [37, 41]. Although reports with different schemes have varied widely, Epstein and Herawi reported an incidence of HGPIN at first biopsy in the order of 5–8%. The median risk for cancer at second biopsy was 24.1% [38]. With a risk no different from that in patients with a benign histology, the authors concluded that immediate repeat biopsy is not necessary [38, 40]. However, De Nunzio and coworkers studied a cohort of 650 men undergoing first-time biopsy and detected HGPIN in 22%. With an overall cancer detection rate of 18.8%, the authors correlated the cancer risk with the number of cores involved and recommended repeated biopsy when more than 4 cores are involved [34]. Furthermore, a recent study by Schoenfield et al. evaluating initial saturation biopsy has also detected a high cancer rate (80%) following the finding of multifocal HGPIN at first biopsy [37].

Borboroglu and coworkers reported an incidence of HGPIN or ASAP of 9.8% in a cohort of 1,391 men undergoing first biopsy. Cancer detection rate at rebiopsy was 47%. Interestingly, the initial biopsy site matched the sextant location of cancer on repeat biopsy in 47%. The authors concluded that having targeted biopsy of the area with HGPIN and/or ASAP would have missed 53% of cancers [42].

Far from definitive, the indications for rebiopsy continue to evolve, as the technique itself does. Most clinicians will offer rebiopsy to patients with persistent clinical suspicion based on persistently high or rising PSA, abnormal DRE, and suspicious TRUS findings. Equally, most clinicians will rebiopsy patients with ASAP, potentially HGPIN, and of course, all those cases with inconclusive histology reports. Implementation of active surveillance protocols, and more recently, a move towards focal therapies has made clinicians reevaluate current practices and explore even more exhaustive schemes such as “saturation biopsy” and also, based on anatomical considerations, a renewed interest on the transperineal approach.

## 6. The Role and Limitations of Saturation Biopsies

In the absence of a standard template for repeated prostate biopsy and with a clear trend towards larger samplings of the gland in the repeated setting, several groups focused the attention on more extensive protocols with the aim to reduce false-negative rates.

 In 2000, Borboroglu and coworkers reported their experience on 57 men with at least one previous negative sextant biopsy (mean: 2.1; range: 1 to 4), who underwent extensive transrectal ultrasound-guided biopsy. Under intravenous sedation, an average of 22.5 cores (range: 15 to 31) was taken. With an overall cancer detection rate of 30%, malignancy was identified in 47% of men with previous HGPIN or ASAP. The authors reported 6 cases of urinary retention and 1 case of rectal bleeding [43]. One year later, Stewart coined the term “saturation biopsy”. In this study, 224 men with previous negative sextant biopsies (range: 1 to 7) underwent an extensive protocol including a mean of 23 cores (range: 14 to 45), reporting an improved cancer detection rate of 34%. The overall complication rate was 12%, with haematuria being the most common event [44]. 

For the last 10 years, the clinical role of “saturation” in the rebiopsy setting has been further scrutinized in several published series, with reported cancer detection rates ranging from 13.5 to 45% [43–52].

De La Taille and coworkers [46] prospectively evaluated the benefit of added cores in a 21-core saturation scheme comprising sextant biopsies at a 45° angle, 6 PZ cores at an 80° angle, 6 TZ cores and 3 biopsies in the midline peripheral zone. With 303 men enrolled, the cancer detection rate using sextant biopsies, 12 cores (sextant plus lateral biopsies), 18 cores (sextant plus lateral plus TZ biopsies), and 21 cores (sextant plus lateral plus TZ, plus midline biopsies) was 22.7%, 28.3%, 30.7%, and 31.3%, respectively. Furthermore, subset analysis correlating positive biopsy cores with final histology in 150 prostatectomy specimens demonstrated higher accuracy of the saturation protocol in predicting T3 disease and positive margins [51]. 

Fleshner and Klotz [45] evaluated a saturation protocol, involving 24 PZ cores, 6 to 12 TZ cores, and 2 transurethral samples, in a highly prebiopsied cohort of 37 men and reported a cancer detection rate of 13.5%, with all malignancies detected in the PZ cores. Rabets et al. performed saturation prostate biopsy in 116 patients with at least 1 prior negative biopsy and reported an overall detection rate of 29%. Interestingly, a 64% detection rate was noted when a patient had undergone a single prior sextant biopsy. They analysed cancer detection rates by type of biopsy and by number of previous biopsies. Cancer detection rates following sextant and ≥10-core biopsies were 41% and 24%, respectively. Cancer detection rate was 33% following single biopsy compared to 24% for multiple biopsies [52]. 

On review of the literature, the question remains of how many cores are optimal on saturation biopsy. Published protocols have incorporated very variable number of cores, usually between 20 to 30, and have reported overlapping results. It seems logical to sample the prostate gland with a sufficiently large number of cores, yet one must attempt to strike a balance with unwanted morbidity. This has been recently evidenced by Simon et al. who utilized an “extensive saturation” biopsy protocol involving a median of 64 cores (range: 39 to 139). With 40 patients enrolled, cancer detection rate was 45%. The authors concluded that there is no significant increase in the cancer detection rate in an extensive saturation-biopsy regimen compared to published series with fewer cores, but the morbidity increased [49]. 

Several studies have proposed that the use of saturation biopsies should not be restricted to patient with previous negative biopsies but should be extended to be used at initial biopsy. However, data have shown that the detection rate using initial saturation biopsies was not significantly increased compared to extended schemes. In 2006, Jones et al. [53] published their results on a cohort of 139 patients (PSA ≥ 2.5 ng/dl) undergoing first-time biopsy compared to those of 87 patients who had previously undergone 10-core initial biopsies. Cancer detection rates were 44.6% and 51.7%, respectively. Complication rates were comparable. The authors concluded that although saturation prostate biopsy improved cancer detection in men with suspicion of cancer following a negative biopsy, it did not appear to offer benefit as an initial biopsy technique. These findings have been further supported by a recently published study by Lane et al. [54], of 257 men undergoing initial saturation biopsy, with a reported 43% cancer detection rate. In the 147 cases with negative initial saturation biopsy, and with a median follow-up period of 3.2 years, the false-negative rate on subsequent prostate biopsy (24%) was equivalent to that following traditional prostate biopsy. 

In the light of these data and those from other groups, it is now widely accepted that saturation biopsy has to be considered only in the repeat setting [30, 31, 55, 56]. Furthermore, even in the repeat setting transrectal saturation biopsy can miss potentially significant cancer in poorly sampled prostate regions, in particular the apical region. Also, the limitations of transrectal sampling need to be considered when planning focal therapeutic approaches, which require a “perfect” spatial delineation of the target tumour. This has encouraged clinicians to pursue transperineal approaches.

## 7. The Transperineal Approach: Definition and Rationale

Far from a novel technique, transperineal needle biopsy of the prostate was first described by Peck back in 1972 [57]. In 1990, Clements and coworkers published a series of 143 consecutive patients with suspicious US and/or digital rectal examination (DRE) and reported a cancer detection rate of 34.3% [58]. However, initial reports were restricted to patients unsuitable for the standard transrectal approach. Several published studies demonstrated the safety and efficacy of transperineal needle biopsy in patients with previous proctocolectomy or abdominoperineal resection [59–61]. While initial series employed transurethral ultrasound guidance [60, 62], Filderman and Jacobs described in 1994 the technique of transperineal ultrasound-guided transperineal prostate biopsy [63]. 

In a comparative study on 20 men with known cancer undergoing biopsy immediately before radical prostatectomy, Shinghal and Terris reported a 10% cancer detection rate with a transperineal US-guided 6-core transperineal biopsy template, compared to a sensitivity of 65% with TRUS-guided sextant biopsies [64]. This result can be in part explained simply by the imaging limitations of transperineal US. Indeed, a recent study has also indicated the technical superiority of TRUS for visualisation and measurement of the prostate gland when compared to transperineal US [65]. Furthermore, the sampling size on that study made the interpretation difficult. In a recent study, Terris and coworkers reviewed 28 patients with elevated PSA (median = 9.5 ng/ml) and previous abdominoperineal resection, undergoing US-guided transperineal biopsies, and reported a cancer detection rate of 82% [66]. The impact of prostate volume and the optimal number of cores in patients undergoing first-time transperineal biopsy has been further emphasized in contemporary series [67, 68].

Despite the initial hesitancy, TRUS-guided systematic prostate biopsies by the transperineal approach have continued to evolve. Indeed, the last decade, driven by the superior sampling of the prostate apex [69] and anterior region [70], has seen an unprecedented renewed interest for these schemes. 

Ficarra and colleagues [67] compared the performance of different transperineal schemes in a cohort of 480 consecutive patients (PSA = 2.5–20 ng/ml) undergoing 14-core TRUS-guided transperineal prostate biopsy, including 12 cores in the peripheral and two in the transitional zone. Detection rates were subanalysed for 14, 12, 10, 8, and 6 cores, by exclusion of pairs of cores and further stratified according to TRUS volume. While the standard sextant biopsies detected 35.2% of cancers, the 8- and 10-core schemes yielded detection rates of 37.1–38.8% and of 39.6–40.8%, respectively. Importantly, with prostate volumes <30 cc, the detection rate of the 14-core scheme (43.8%) was not statistically different to that obtained with the 8-peripheral core protocol. In patients with 30.1–50 cc prostate volume a 12-peripheral core scheme was equivalent to a 14-core sampling. Above 50 cc, even the 14-core scheme (24.2%) was found to be insufficient, the authors recommending larger number of cores for larger glands. 

Several series evaluating different transperineal schemes in the first-time biopsy setting have been published in the last 10 years, reporting cancer detection rates in the order of 18.8–72.1% and 24–75.4%, when employing 6 and 12 cores, respectively [68, 71–75]. More extensive schemes have also been evaluated in this setting, reporting cancer detection rates of 36% with 14 cores [76] and up to 49% with 18 cores [77, 78].

 Rocco and coworkers at the European Institute of Oncology in Milan evaluated the sensitivity and detection rate of 12-core transperineal biopsies in 63 patients not previously investigated for prostate cancer (median PSA = 1.2 ng/ml), undergoing radical cystoprostatectomy for bladder cancer. In this cohort, 17.2% of biopsies were positive, resulting in an overall sensitivity of 32.3%. However, sensitivity for clinically significant cancers was 75% [79].

## 8. Comparison of Transperineal and Transrectal Biopsy Schemes

Few studies have directly compared the performance of the transrectal and transperineal approaches in the sextant and extended settings. In an *ex vivo* study, Vis et al. compared US-guided sextant transverse (transrectal) biopsy and subsequent sextant longitudinal (transperineal) biopsy on 40 radical prostatectomy specimens. Of 40 cancers, 82.5% were redetected by the transperineal and 72.5% by the transrectal biopsies. The authors suggested an improved sampling of the PZ with the transperineal approach [80]. A contemporary prospective clinical study by Emiliozzi et al. in which 107 patients underwent combined TRUS sextant and 6-core “fan” transperineal biopsies, reported a cancer detection rate of 32% and 38%, respectively. The transperineal scheme detected 41/43 (95%) of the cancers in the cohort [81]. However, other published series comparing first-time biopsies employing extended 12- and 14-core templates have not found significant differences between the two approaches [82, 83]. Takenaka et al. in a prospective randomized study comparing the diagnostic efficacy of transperineal and transrectal 12-core biopsy, found similar overall cancer detection rates. However, the transperineal approach was superior in the subset of patients with PSA in the 4.1–10 ng/ml range [84].

## 9. Transperineal Biopsy Schemes in the Repeat/Saturation and Prostate Mapping Settings

The above studies have highlighted the importance of improved tissue sampling by targeting the anterior prostatic apex, the far-lateral peripheral zone, and in particular cases, the transitional zone. Further evidence has been derived from recent computerized models [85] and published series of saturation protocols, involving larger number of cores [35, 70, 77, 86, 87]. These approaches have demonstrated their superiority in the repeated setting, where one or more previous biopsies have failed to detect occult cancer. 

In 2001, Igel et al. [88], reported their experience in 88 men with clinical suspicion of prostate cancer and, at least, one previous negative biopsy. Systematic transperineal biopsy detected cancer in 43% of cases, with involvement of the TZ in 76% of cases. 

Since that date, several studies on transperineal saturation schemes have been published, reporting cancer detection rates of 22.7 to 43% [86, 88–93]. With variable number of cores, usually 20 to 30, these schemes have still detected cancer even in highly prebiopsied cohorts. Furthermore, a recent study by Li et al. [94] has evaluated an 11-region transperineal saturation template in 303 with clinical suspicion of prostate cancer in the first-time setting. With a mean of 23.7 cores (range: 11 to 44), the reported overall cancer detection rate was 37.6%. Stratification by level of PSA resulted in cancer detection rates of 22.2%, 8.2%, 21.6%, 48.4%, 68.4%, and 100% for PSA levels of 0 to 4.0, 4.1 to 10.0, 10.1 to 20.0, 20.1 to 30.0, 30.1 to 70.0, and >70.1 ng/mL, respectively.

Importantly, improved early cancer detection has resulted in a down-stage shift which, paired with the development of novel technologies and energy delivery systems, has translated in a rapid acceptance of focal therapy of prostate cancer as a viable therapeutic option. This has been further supported by recent evidence that up to 20% of prostate cancers will be completely unilateral and candidates for focal ablation [95, 96]. However, the application of focal therapy demands a rigorous patient selection based on a reliable and accurate tumour-target location. In this setting, several recently published studies have highlighted the limitations of sextant [97] as well as extended transrectal prostate biopsy schemes [95, 98, 99].

Mayes et al. [97] evaluated the reliability of routine sextant biopsy to detect unilateral lesions in a cohort of 365 men who subsequently underwent radical prostatectomy (RP). When the sextant biopsy detects unilateral disease, according to RP results, the NPV was high (91%) with a low false-negative rate (9%). However, it has a PPV of 28% with a high false-positive rate (72%). In agreement with other earlier [100] and contemporary [101] studies, the authors concluded that routine sextant biopsy cannot provide reliable, accurate information about the unilaterality of the tumour.

Tsivian et al. [95] recently published a retrospective analysis of 882 biopsy-proven cancers (729 sextant, 153 extended biopsies) comparing sextant versus extended 12-core biopsy protocols. The sensitivity (from 84.1% to 88.0%) and specificity (from 37.1% to 53.9%) improved on sextant and extended biopsy, respectively. With a decrease in false-positive rates from 62.9% to 46.1% and false-negative rates from 15.9% to 12%, the overall diagnostic accuracy increased from 49% to 59%, respectively. However, the authors concluded that, although superior to sextant biopsy, a 12-core scheme is not an ideal diagnostic test to select patients for focal therapy. 

On subsequent analysis of this cohort [102], including 859 patients and substratification by prostate weight (≤40 and >40 g) and biopsy scheme (6–9 sextant and 10–20 cores extended), unilateral disease was more common in prostates >40 g both on biopsy (69% versus 60%) and on final pathology (21% versus 14%). In this study, extended biopsy protocols performed better than sextant but the benefit was statistically significant only in prostates >40 g. 

In response to modern diagnostic and therapeutic requirements, the transperineal approach has further evolved towards a sophisticated staging procedure. Improved prostate sampling can be further optimized by the use of a fixed brachytherapy grid, allowing a rigorous and reproducible 3D mapping of the gland (3D-PMB) [103, 104]. “Solid” 3D computer models reconstructed from autopsy and T1c prostatectomy specimens have demonstrated a superiority of the 5 mm grid in cancer detection (75% versus 33%) when compared to the 10 mm grid [105]. 

In 2008, Onik and Barzell [106] reported their experience on 110 men with unilateral disease on TRUS biopsy, undergoing restaging using the 3D mapping scheme prior to focal therapy. With a median of 46 cores (SD ± 19) taken every 5 mm, this scheme detected bilateral cancer in 55% and upgrading in 23% of cases. Updated results, including 180 cases, were published in 2009. With a median of 50 cores (SD ± 20.6), bilateral disease was identified in 61.1% of cases, with a 22.7% increase on Gleason score. Complications were self-limited and included a 7.7% incidence of acute urinary retention [107]. 

An alternative approach, aimed to exploit the advantages of both, the transrectal and the transperineal biopsy schemes, has recently been developed and is currently under evaluation. 

In 2005, Watanabe et al. [108] evaluated a 12-core extended protocol involving a standard transrectal sextant biopsy plus a 6-core (lateral PZ, parasagital PZ and TZ) transperineal template. The reported overall detection rate of 48.5% was found to be superior to either transrectal or transperineal templates alone. 

Since 2006, Kihara's group at the Tokyo Medical and Dental University [109–112] have extensively evaluated a more extensive sampling of the prostate combining 12-core transrectal and 14-core transperineal biopsy templates (3D26PB), demonstrating potential application in the staging of patients prior to radical surgery. Although there was no significant improvement in men with abnormal DRE (22% improvement; *P* = .18), cancer detection was significantly improved by 85% in men with normal DRE (*P* = .0004) utilizing this technique, suggesting a potential diagnostic advantage of the 3D26 biopsy over the transrectal biopsy to detect stage T1c disease in men with normal DRE.

## 10. MRI-Guided Transperineal Biopsy

With the rapid technological development of MRI and building on the experience acquired with MRI-guided brachytherapy, investigators at the Brigham and Women's Hospital introduced an MRI-guided prostate biopsy program, employing a transperineal approach, with direct real-time image-guided sampling of the prostate and any suspicious lesions [113–115]. The program has successfully been performed by this group in over 50 biopsy cases, reporting a cancer detection rate of 30% [116]. Simultaneously, a group from the Johns Hopkins University and the National Institutes of Health have also explored this novel technology and performed transperineal biopsy in a series of eight procedures in four patients, to perform a total of 32 targeted biopsy needle placements within the prostate.With a mean biopsy needle placement error of 2.1 mm, 95% of the needle placement errors were less than 4.0 mm, demonstrating the accuracy of modern MRI scanning in targeting prostatic lesions [116]. This error can be further reduced with the routine use of needles with a symmetrical bevel, as demonstrated by Blumenfeld et al. [117]. MR-guided biopsy, due to its high sensitivity and higher specificity than US, has proven to be a useful alternative to US-guided procedures [118]. MRI guidance allows high spatial resolution and multiplanar volumetric imaging capabilities, which can be exploited in selection and planning of focal therapies, further enhancing the advantages of the transperineal approach.

## 11. Conclusion

In this paper, we have attempted to summarise the interesting evolution of the refinements to prostate biopsy since Hodges and colleagues, in 1989, first demonstrated the superiority of systematic TRUS biopsies, compared to digitally directed biopsy. However, sampling limitations of this approach, strongly evidenced with the wide introduction of PSA testing, moved the clinicians to embrace more extended approaches. Certainly, a 10–12 core systematic biopsy, targeting the far lateral aspect of the peripheral zone, has now become standard practice for initial biopsy. However, these extended schemes have been demonstrated to be insufficient in the repeat biopsy setting. With persistent clinical suspicion and initial negative sampling, saturation approaches are now the preferred option by the modern clinician, although data have demonstrated a limited value at initial biopsy. Saturation biopsy has been favoured via a transrectal approach as this can be performed in the office setting, under local anaesthesia and mild sedation, with few side effects. Nonetheless, the transrectal approach has limitations in sampling the anterior regions of the gland, in particular the apical segment. These sampling restrictions, together with the rapid development of minimally invasive and in particular focal therapies, have driven a renewed interest in the transperineal approach. Despite the requirement for general anaesthesia and a potential increased urinary retention rate, novel transperineal mapping schemes, when employing a brachytherapy grid template, allow for more accurate sampling of the entire gland. The remit of prostate biopsy now lies beyond pure diagnostics and has become an essential tool for determining the optimal therapeutic approach. A comparative trial of biopsy strategies using step-sectioned radical prostatectomy specimens should answer outstanding questions and define the future in this important area.
